# Disentangling Jenny’s equation by machine learning

**DOI:** 10.1038/s41598-023-44171-x

**Published:** 2023-11-27

**Authors:** F. Prieto-Castrillo, M. Rodríguez-Rastrero, F. Yunta, F. Borondo, J. Borondo

**Affiliations:** 1 Departamento de Matemáticas, Universidad de Oviedo, Calle García Lorca 18, 33007 Oviedo, Principado de Asturias Spain; 2 Departamento de Medio Ambiente, Centro de Investigaciones Energéticas, Medioambientales y Tecnológicas (CIEMAT), Avenida Complutense 40, 28040 Madrid, Spain; 3 Joint Research Centre (JRC), European Commission, Via Enrico Fermi 2749, 21027 Ispra, Italy; 4 Departamento de Química, Universidad Autónoma de Madrid, 28049 Cantoblanco, Spain; 5 Departamento de Gestión Empresarial, Universidad Pontifícia de Comillas, Madrid, Spain; 6 AgrowingData, Almería, Spain

**Keywords:** Biogeochemistry, Solid Earth sciences, Mathematics and computing

## Abstract

The so-called soil-landscape model is the central paradigm which relates soil types to their forming factors through the visionary Jenny’s equation. This is a formal mathematical expression that would permit to infer which soil should be found in a specific geographical location if the involved relationship was sufficiently known. Unfortunately, Jenny’s is only a conceptual expression, where the intervening variables are of qualitative nature, not being then possible to work it out with standard mathematical tools. In this work, we take a first step to unlock this expression, showing how Machine Learning can be used to predictably relate soil types and environmental factors. Our method outperforms other conventional statistical analyses that can be carried out on the same forming factors defined by measurable environmental variables.

## Introduction

In 1960 the Nobel Prize in Physics Eugene Wigner published a fascinating paper^1^ on *The unreasonable effectiveness of mathematics in the natural sciences*. Despite the generality of the title, the text was mainly restricted to physics, but the belief that mathematical equations are the best way to go in translating relationships and interactions has always been at the deepest root of science.

Other disciplines, like biology or geology, have traditionally kept outside this stream, by accepting paradigms consisting of a seemingly endless resort to a multiplicative use of taxonomy, trying to cope with the tremendous diversity in the subject. This situation suffered a dramatic change in biology at the turn of the century, when the appearance of complex networks theory^2,3^ brought long-awaited tools to help tackling some of their problems^4,5^; this being also true in sociology^6,7^. The new approach shifted the focus from diversity to the web of interactions among species or individuals. This approach became even more successful with the advent of Machine Learning (ML).

**Figure 1:**
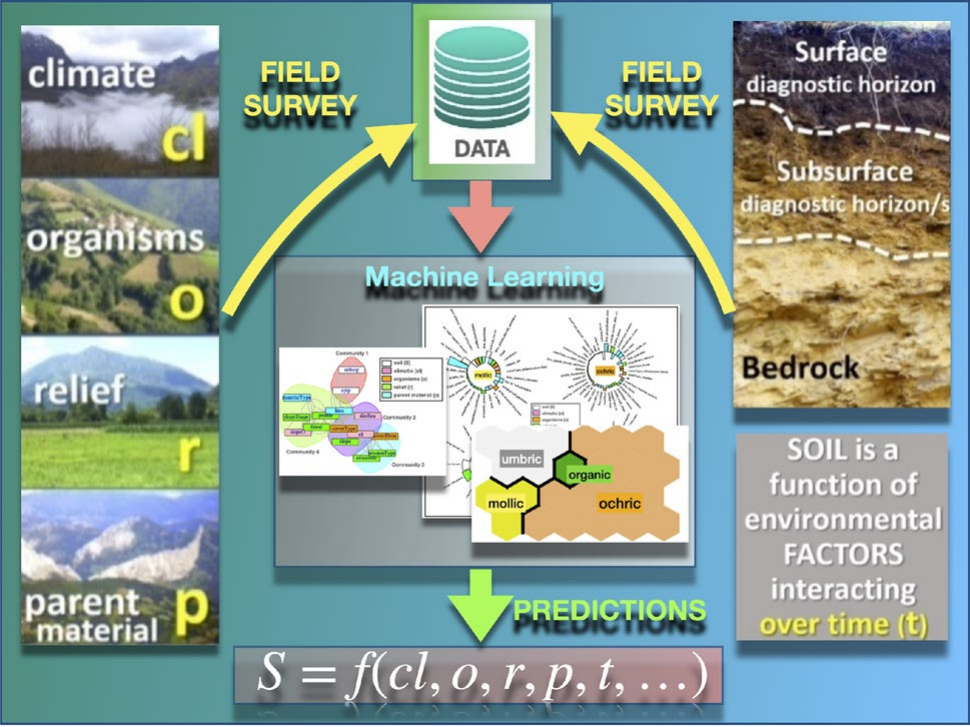
The Jenny equation^11^ relates the soil concept, *S*, to the forming factors: climate (*c*), organisms (*o*), relief (*r*), and parent material *p*, which interact over time *t* within the soil-landscape paradigm of Soil Science. Unfortunately, the function $f(\ldots )$ cannot be defined in standard mathematical terms, since the involved variables are often categorical, this preventing its use in a straight direct way. Jenny’s equation is an effort to establish the form of $f(\ldots )$. Modern techniques in Machine Learning provide adequate algorithms formally defining this function by extracting knowledge from large data sets formed by different magnitudes characterizing the soil forming factors, in a way able to make accurate predictions.

Similarly, in Soil Science, understanding soil state-and-change in response to different natural or humans factors still remains a great, yet important, challenge^8^. The so-called *Soil-Landscape Model*, graphically described in Fig. 1, is the operational paradigm^9^ on which field surveys are based. The model assumes that the soil state is a function of the complex interaction of some (landscape) forming factors^10^, which creates a pattern of layers, on the decimeter scale, more or less parallel to the earth surface, called soil horizons^11,12^.

The classical denomination of soil horizons relies on remarkably subjective criteria imprinted by different researchers, something that motivated the introduction of the so-called ’diagnostic horizons’, based on measurable physical, chemical, and morphological properties^12,13^, aiming to establish a classification (soil taxa) . Diagnostic horizons reflect soil properties in a simpler way and they are susceptible to spatial representation^14^.

In this scenario, first Dokuchaev and later Jenny^11,15^ developed a seminal milestone of the paradigm, proposing a formalization of the soil forming factors, in the form of the famous *clorpt* mathematical expression

1 $$\begin{aligned} S= f(cl, o, r, p, t,\ldots ). \end{aligned}$$

In it, *S* is a currently existing (local^16^) soil, expressed either as a specific taxon or as one of its diagnostic horizons. On the r.h.s. of the equation, *cl* (atmospheric climate), *o* (organisms), *r* (relief and landforms) and *p* (parent material) constitute spatially located environmental factors^17^, and the time factor, *t*, indicates the duration of the interaction among them^18^. Had all symbols in Jenny’s equation have a unique and precise numerical meaning, this equation would have been the central computational expression in pedology^19^, surely what Dokuchaev and Jenny originally had in mind in their visionary approach. But this is not the case. To make things worst, Eq. (1) does not specify which raw variables integrate the forming factors; however, we know, for example, that *cl* must be an aggregate of other variables (altitude, distance to the sea, etc.), which have to be considered, nevertheless, context dependent. Therefore, the *clorpt* signature in the argument of $f(\ldots )$ turns out to be a template or coarse-grain vision that has to be explicitly specified. This is also true for the l.h.s. of the equation, since the soil type is, in general, a combination of epipedon and endopedons.

However, despite the fact that Eq. (1) is more of a descriptive expression for being all involved variables of qualitative synthetic type, and *f* impossible to formulate with standard mathematical expressions, we are now in an era where the development of ML can provide tools capable of turning Eq. (1) into a true predictive device. Modern techniques in ML can extract knowledge from large data sets (soil surveys), thus supplying the adequate algorithms to make of Jenny’s function *f* an algorithmically defined one.

The aim of this paper is to contribute to narrow the gap between Pedology and Mathematics, by developing a method, based on the application of self-organized maps (SOM)^20,21^ to ground-truth soil data, able to quantitative compute relationships between soils and their environmental forming factors. More specifically, we show here to what extent a ML approach can be defined that represents a first step towards a true reinterpretation of Jenny expression (1) that: i) is coherent with the empirical knowledge provided by field work, and ii) it can be established in simple mathematical terms/model.

Embedded in this philosophy is also digital soil mapping (DSM)^22,23^, a very interesting application of (1) which in recent years have witnessed an increasing demand due to potential applications. Using neural networks or other deep learning techniques, DSM aims at predicting soil classes or attributes at unvisited geographical positions by their relationship with environmental covariates. In this way, the distribution of valuable resources^24^, or soil types in extensive geographical regions, such as France^25,26^, Italy^27^, or southeastern Brazil^28^ have been studied in the last years.

Figure 1 summarizes the concepts related to the soil-landscape paradigm used in the present work in a pictorial way.

**Table 1:** Dataset variables description, indicating name and synthetic description, relationship with the symbols in Jenny’s equation (1), type, and range of values (numerical) or number of categories per variable (categorical).

Symbol in Eq. (1)	Variable	Description	Type	Levels	Values
*S*	*epip*	Diagnostic surface horizons	Factor	4	Mollic; ochric; umbric; organic
*S*	*subsup*	Diagnostic subsurface horizon	Factor	5	Cambic-albic-no; albic-umbric-spodic-no; argilic-no; calcic-no; no-no
*cl*	*alt*	Altitude (a.s.l.)	Numerical	Range	[0, 1957] (m)
*cl*	*distSea*	Distance to closest shore	Numerical	Range	[0, 69] (km)
*cl*	*slopeO*	Slope aspect (exposure)	Factor	9	N; NE; E; SE; S; SW; W; NW; no
*o*	*coverType*	Vegetation cover type	Factor	8	Agricultural forage crop; natural woodland; eucalyptus forest; grassland meadow; bush; grassland bush; pine reforestation; meadows
*o*	*coverDens*	Vegetation cover density	Numerical	Range	[0, 100]
*r*	*profile*	Hill slope profile position	Factor	6	Summit; doline; flood plain; high slope; low slope; flat
*r*	*slope*	Slope value	Numerical	Range	[0, 175]
*r*	*slopeShape*	Slope shape	Factor	4	concave; concave-convex;
					Convex; rectilinear
*r*	*flood*	Flooding or ponding potential	Numerical	Range	[0, 2]
*r*	*erosionType*	Erosive forms	Factor	3	Landslide; groove; no
*r*	*erosionSt*	Erosion intensity	Factor	3	Strong; weak; no
*p*	*materialType*	In situ vs. transported	Factor	2	In-situ; deposit
*p*	*litos*	Type of parent material (bedrock)	Factor	11	alluvial glacial sediment; mixed siliceous; clays; sands; sandstones; limestones; decalcification clays; shales; quartzites; organic material; lutites

The endopedon variable *subsup* is the combination of the diagnostic subsurface horizons 1 and 2 ($subsup_1$ and $subsup_2$, respectively). See full details in “Methods and auxiliary calculations”.

## Results

The final results obtained with our ML analysis, performed according to the objectives stated above, are presented in this section. As shown below, our dataset contains sufficiently pedogenetic relationships between different surface and subsurface diagnostic horizons to grant a convenient way to evaluate the effectiveness of the obtained ML models. Indeed, our analysis has revealed non-trivial associations between the forming factors.

However, due to the limitations of prospecting and data acquisition, intrinsic to any soil survey, the factors have an heterogeneous level of description, our data containing few observations for some variables. We face therefore in this work a multi-dimensional analysis with heterogeneous variables, incomplete data, and almost surely non-linear relationships between them. An appropriate way to address this type of problem is through clustering techniques and dimensionality reduction.

### Definition of variables in our dataset

The dataset used in this study consists on the observed values for the 15 variables related to the symbols in Eq. (1), listed in Table 1, that were collected in an extensive field study carried out in the Principado de Asturias (Spain). The variables have been chosen to be relevant in a context-dependent sense in a region that, although relatively reduced in geographic terms, shows a wide variety of soil types. However, no measurable variable was assigned to the forming factor *time*. It has been kept constant in this work since its effect can be explained by the other soil forming factors. More details are given in  “Methods and auxiliary calculations” and “Conclusions”.

Each observation point can be classified according to one or more diagnostic horizons, which in turn can be either superficial (epidedon/topsoil) or subsurface (endopedon/subsoil), as shown in Fig. 1.

Now, it is desirable a simplification in the variables on both sides of Eq. (1), so that the soil variables and forming factors are presented in the form of an aggregated set of (numerical and categorical) variables, while preserving, at the same time, their basic pedological meanings.

**Figure 2:**
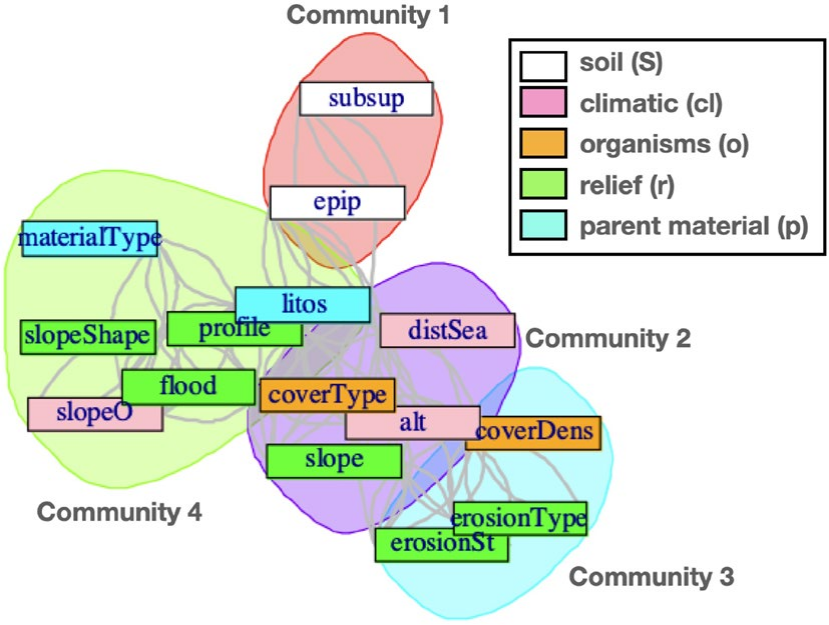
Clusters of variables in Jenny equation (listed in Table 1) obtained by applying a median cutoff on the distances between variables and a community short random walk detection algorithm (see Fig. 11 in “Methods and auxiliary calculations” for details).

### The most relevant associations among forming factors

For that purpose, we perform first a preliminary exploratory analysis of the data in order to unveil the existence or not of possible non-trivial correlations between the variables involved in Jenny’s equation. The procedure is as follows. Starting from the complete graph consisting of the 15 forming factors in Table 1 as vertices, we first prune it, by removing all edges connecting nodes lying at a distance, computed with Eq. (2), greater that the associated graph median. Then, the existing network communities are identified using a short random walk technique^29^ (see full details in “Factor association: exploratory analysis”).

The result is shown in Fig. 2, where an interesting structure, consisting of four differentiated clusters, is clearly observed.

The first one, namely that marked in red at the top-right corner, is very obvious *a priori*, since it aggregates the epipedon (*epip*) and endopedon (*subsup*) variables, defining the l.h.s. of Jenny’s expression. This indicates that, from the ML point of view, they all act as valid proxies of the soil type *S*. In this way, we can use from a practical point of view the observed epipedon as a proxy of the soil state, *S*, which can be: ochric, mollic, umbric, or organic, a variable whose number of categories is very limited (only 4) compared to *taxa* (with a potentially unlimited number of categories). Let us also remark aside that this is more suitable for remote sensing analysis, and is consistent with the basis for the two biggest European soil datasets LUCAS and GEMAS^30,31^.

The other three communities define the interactions among the variables in the r.h.s. of the equation, and they are not so easy to interpret, since they clearly depart from the *naive* conception solely in terms of the *clorp* signature. However, our result clearly indicates that they come in groups, and some interesting observations can be extracted from this fact. First, the *p* (parent material) variables only appear in the green community, mixed with slope related variables, *r* and *cl*. Second, no *cl* (climate) variable participates in the cyan cluster, which aggregates erosion and cover density variables, belonging to *r* and *o*, respectively. Finally, the purple community consists of a more heterogeneous mix of characteristics. This result reinforces our hypothesis that the forming factors in the Jenny equation are actually non-trivial aggregations of observables, i.e., those appearing together in the same cluster in Fig. 2.

In order to complement this analysis and further interpret the resulting network, we have computed partial correlations. The corresponding results are shown in Appendix D of the Supplemental Material (SM) S1, where we discuss the significant relations found with this indicator, and compare them with the results of Fig. 2.

### Soil-profile signatures for endopedon vs. epipedon

**Figure 3:**
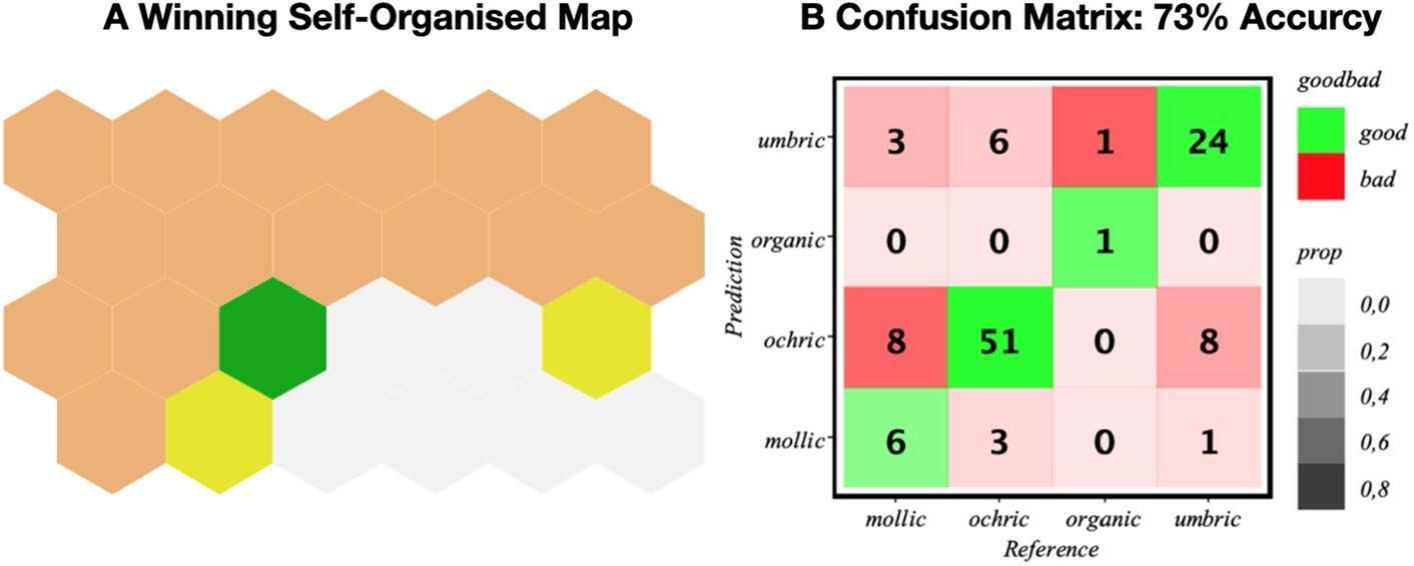
Multi-SOM model obtained from the field survey dataset with the iterative procedure described in “Model building”. (**A**) SOM-map showing the aggregated classes in the diagnosis horizons layer; (**B**) Confusion matrix and accuracy of the optimal model.

The previous result clearly discourages from attempting simple linear, or similar, regression type models to tackle expression (1). Having this result in mind, we further extend our research by using a multi-SOM map^20,32–34^ (see “Model building” for details).

**Figure 4:**
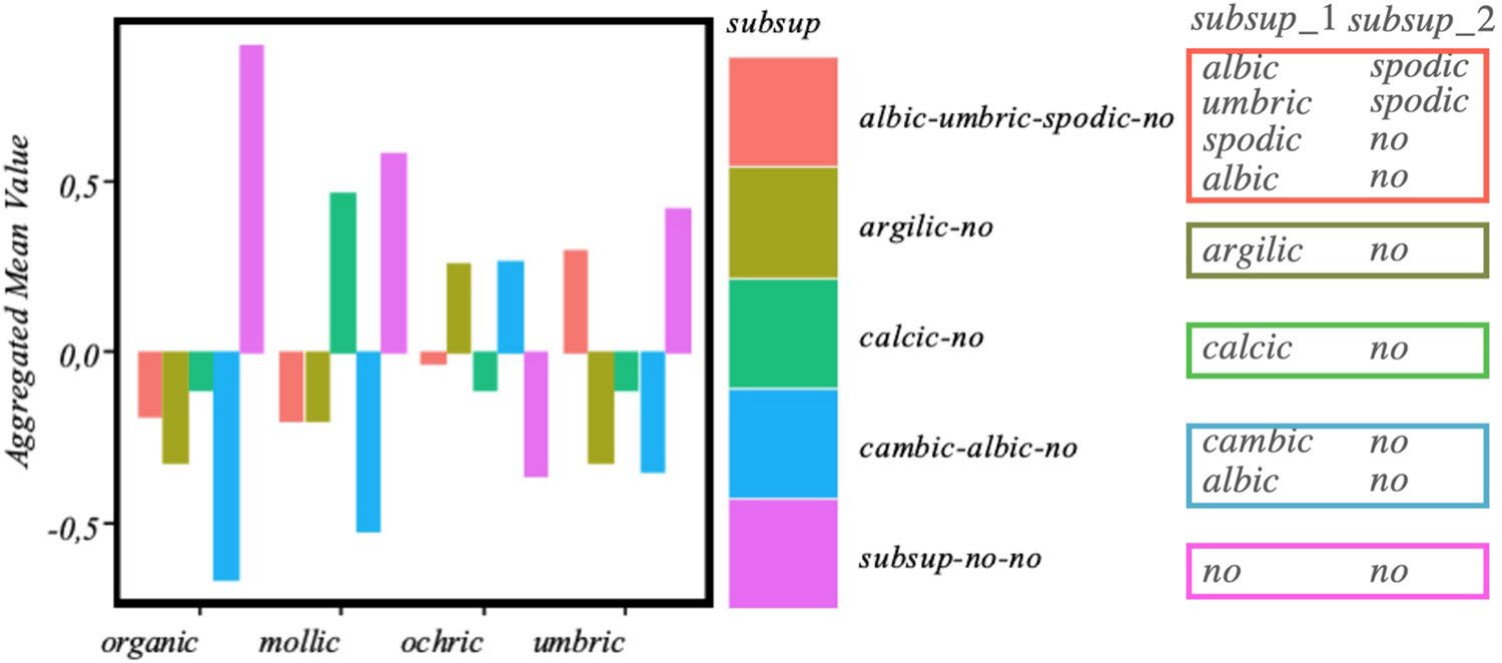
Results from the multi-SOM model with the soil-profile signatures for endopedon *vs.* epipedon. Each bar represents the mean value of the stardarized (z-scored) *subsup* in each SOM cluster shown in Fig. 3. Positive (negative) heights represent values for that quantity above (below) the mean in the correspondig SOM cluster. The values of the combined variable *subsup* are also shown in the right panel. For example, the *subsup* value $cambic\_albic\_no$ means that the two subsoil observations can achieve values $subsup\_1=cambic$ and $subsup\_2=no$ or $subsup\_1=albic$ and $subsup\_2=no$.

The corresponding results, using a map of 24 neurons, are shown in Fig. 3, where we see how the map is able to detect the four diagnostic horizons existing in the dataset, with a fairly good prediction accuracy, which averages to 73% [see panel (B)] when the model is run many times. Some realizations, nevertheless, can achieve up to 80% accuracy, probably due to a fortunate random split between training and test data. Here, we adopt the conservative position in which we will take the worst-case scenario. Moreover, the confusion matrix of panel (B) reiterates the good predicting accuracy of our map, except for the case of the umbric and mollic epipedons which tend to be confused with ochric ones. This result is not expected as has been discussed, for example, in Ref. ^35^. Another interesting finding extracted from the SOM map analysis concerns the relationship predicted by our model between epipedon and endopedon variables, this including the case when the later is/are missing. The results are shown in Fig. 4, where a rather simple correlation is observed, i.e. organic soils appear mainly associated with $subsup=no\_no$ (both $supsup\_1$ and $subsup\_2$ missing), mollic with $subsup=calcic\_no$ ($supsup\_1=calcic$ and $subsup\_2$ missing), umbric with $subsup=albic\_no$ ($supsup\_1=albic$ and $subsup\_2$ missing), and the ochric, which is the most complicated [also responsibe of more mistakes (see Fig. 3B)], is associated with $subsup=no\_no, argilic\_no,$ and $cambic\_no$. This indicates that either epipedons or endopedons -or a combination of both- can provide a valid proxy for soil characterization. This conclusion is further reinforced by the fact that the *subsup* variable is very weekly connected with the rest of variables in our model, actually only with *litos*, as can be seen in Fig. 2. Our previous hypothesis, that the epipedon is a valid signature to predict soils, is then supported by the analysis.

### Optimal forming factors for each epipedon

A similar analysis can be performed for the forming factors, but in this case we take one step further in interpreting the results. In particular, we address the issue of the possibility of disregarding a significant portion of variables in Table 1, and still maintain the good predictive performance of our model, something that would make of Jenny’s a more workable and easy to interpret mathematical expression.

**Figure 5:**
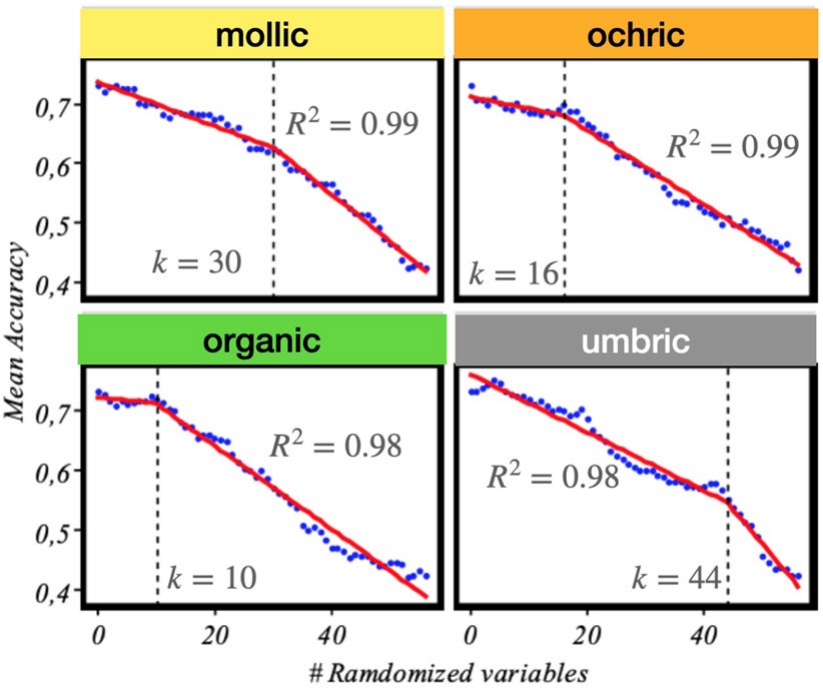
Optimal minimum number of *clorp* variables characterizing each epipedon in Jenny’s equation (1). It is interesting to see how epipedons *need* different subsets of variables and can be oblivion to others.

**Figure 6:**
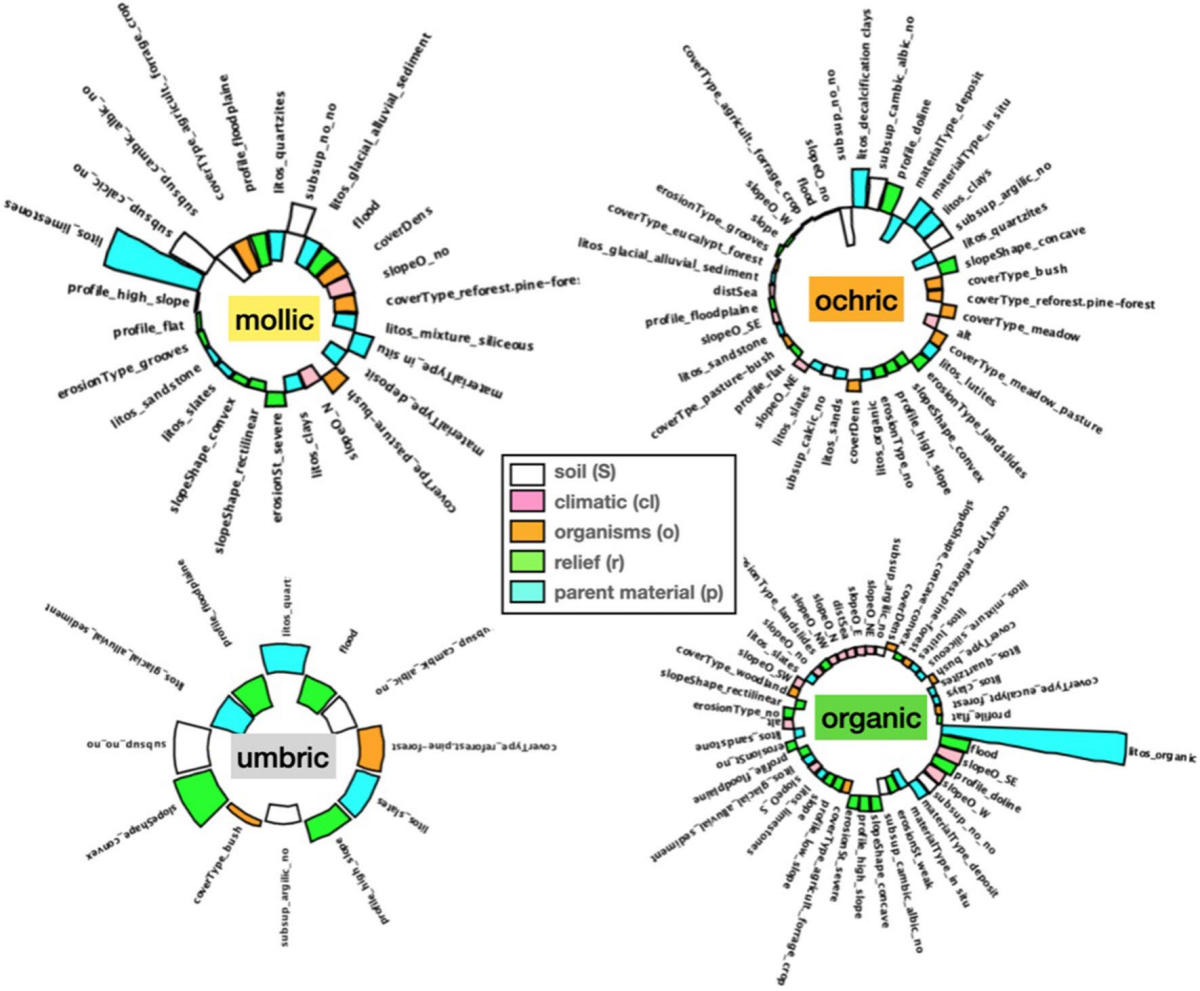
Relative importance of the optimal forming factors for each epipedon. Sorted standarized values of the subset of variables in Table 1 which are optimal for each epipedon according to the method described in “Key forming factors detection”. These results constitute the soil-profile signatures for the key forming factors A negative value here indicates a variable which ranks below the mean in its multi-SOM cluster.

For this purpose, we perform a permutation importance analysis^36^. The full procedure, described in detail in “Key forming factors detection”, essentially consists in ‘eliminating’ variables, one at a time, by making them random, and simultaneously monitoring the loss of accuracy. That is, once the variables have been ordered by their importance in the SOM, we randomize a set of variables of increasing size, *k*, starting with the least important variables in the list. With this set, we run the model again and measure its predicting accuracy. The optimal value of ‘eliminated’ variables can be found by fitting the values to a bilinear model with a varying elbow point^37^. The corresponding results are presented in Fig. 5, where we plot for each epipedon/cluster found in the SOM the averaged accuracy values computed from 150 samples for each random subset. This optimal *k* value can be found by fitting the values to a bilinear model with varying elbow point (vertical dashed line) and then minimizing the error, as described in^37^. It is interesting to see that these sets are in general different; for example for the organic horizon the prediction accuracy regime changes to worse after 10 variables have been eliminated, and then we need to retain 46, while in the umbric case up to 44 variables have to be eliminated to predict the threshold, being then left with only 11. This constitutes a clue to find the meaningful relationships between forming factors and soil in the Jenny equation (1). Finally, for each optimal set of variables, the model is run again to extract the mean values of each variable in the different SOM classes, i.e., epipedons. This allows to interpret which are the most relevant values that characterize each epipedon, which is equivalent to ‘solve’ Jenny equation for our dataset.

The corresponding results are shown in Fig. 6, where the relative importance of the connections existing among epipedons and associated *clorp* variables, as quantitatively ascertain from the dataset by our SOM model, is plot in the form of circular histograms. Notice that, since variables in the multi-SOM are standardized with zero mean, a negative value here means that the corresponding variable ranks below the mean computed in its multi-SOM cluster. The results in this figure constitute the soil-profile signatures for the key forming factors. A close examination of the results show that in all cases, the scenario is mostly controlled by a few variables. The most obvious case is the umbric horizons, where it can be seen that only 6 above and 5 below mean variables are the most relevant, being the rest negligible. And this also true for the rest, if one is ready to give up some accuracy. The worse case being the mollic horizon, for which reducing the forming factors to 6/5 implies a loss of accuracy of just a mere 3.4% (see Fig. 5).

To complement our study, and further interpret our model and the importance of each forming factor in the Jenny’s equation, we have carried out an analysis using the Shapley values method^38,39^. Shapley values provide a measure of the importance of each forming factor by estimating the relative contribution of each factor. The corresponding results are reported in the Appendix E of the SM. In general terms, they are in agreement with the results of our permutation importance analysis, but they fail to discriminate which factors are more important for the *epipedon*.

**Figure 7:**
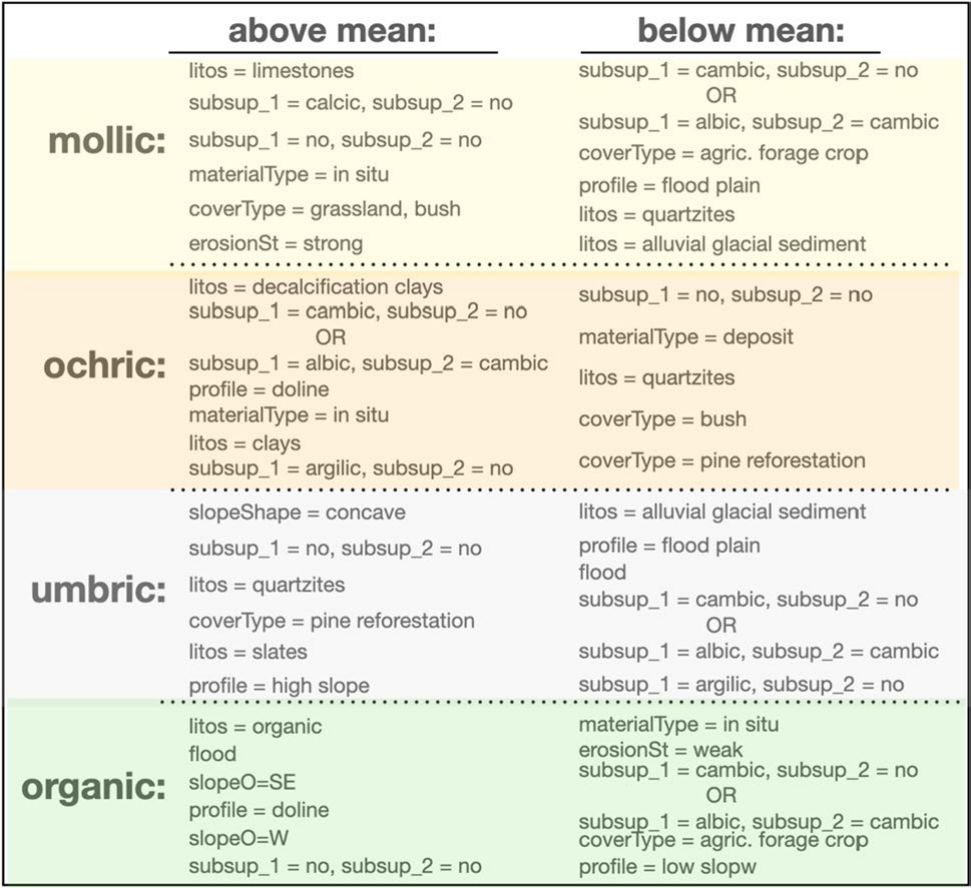
Final variable selection. Six/Five most important variables with average values above/below the mean in each SOM cluster obtained from the results shown in Fig. 6 as a final reference to characterize epipedons in an operational way. Within each epipedon, the variables are ordered (from top to bottom) by importance.

To conclude, we take the 6/5 pattern discussed above into account, and list in Fig. 7 the most relevant selected variables from Table 1, as a final reference to characterize epipedons in an operational way. Notice how our analysis has reduced the number of forming factors necessary to fully understand Jenny’s seminal equation in a very substantial way, this being the most important result of our work.

## Discussion

In this paper we developed a computational procedure able to effectively determine the relevance of the different variables associated to each of the symbols in the Jenny equation (1), as well as to uncover the structure of the dependencies between them, in order to elucidate an operational expression or algorithm towards the automatic characterization of soils. In other words, we have taken a first step to pave the road making of Jenny equation a real quantitative and objective expression, which application does not require the educated analysis and interpretation of field data by soil science experts.

No variable has been selected to represent *time*, a key aspect of Jenny’s equation and pedogenesis. Time factor is not directly recognized in soil taxonomy. The large paleo-group is set up in soil orders such as Alfisols, Mollisols and Ultisols, where it points out to the presence of horizons with an intense accumulation of alluvial clay, whose genesis is associated in part with the soil age but also with the climate. Podzols can be explained by using *time* as forming factor from soil types such as ultisols, but they can also be explained by using other forming factors. In this work, *time* has been taken as constant, as its cartographic representation is very difficult, and it can only be laid down as forming factor in genetically related soils that evolved under similar forming factors, such as vegetation, climate and relief^40^.

For the purpose of the paper, a simple analysis of communities was first carried out. From the results, reported in Fig. 2, we can first conclude that there exist different clusters of variables in Table 1 indicating that certain combinations of soil characteristics behave as groups. In this sense, a cluster containing only the epipedon (*epip*) and endopedon (*subsup*) variables has been found, which strengthens our confidence that our method is correct. Second, the associations found by our analysis for the other variables are not always obvious. For example, the fact that distance to the sea (*distSea*), altitude (*alt*), slope (*slope*), and cover type (*coverType*) behaves as an independent community (see Fig. 2) leads us to believe that the geographic-climatic factors combine with the specific organic factors to form a complex accurate predictor. The same conclusion applies to the other two communities found in the analysis. These two results suggest the existence of strong interactions between the variables, that argue against the simple linear models often used in the literature.

For this reason, we also developed a more sophisticated supervised classification ML model, based on a multi-layered SOM. With this approach a good accuracy rate was achieved, as shown in Fig. 3B, despite the scarcity of the data sample used. This result allows us to claim that the aggregation of neuron into the clusters obtained by the SOM learning process is a reasonable proxy for the joint probability distribution in the data. Indeed, as each cluster in the epipedon layer maps into many neurons in the numerical layer, the resulting SOM allows to establish which factors are most important for each epipedon. In particular, we have obtained in Fig. 4 quantitative relationships between the different subsoil classes, i.e. values of the two layers $supsup_1$ and $subsup_2$, that were far from trivial. For example, we observed that the organic, mollic and umbric classes prevail for those profiles lacking a subsoil layer, while the ochric class is more frequent for soils with a first argillic and cambic-albic sublayer. We stress that other relationships in Fig. 4 were previously observed^40^. This confirms our ML model and therefore validates the reported groupings of factors into epipedon classes.

To strengthen our understanding of the relationship of the factors in Jenny’s Eq. (1), we have proceeded in a systematic way by gradually eliminating variables in Table 1 while simultaneously controlling the predictive accuracy of our model. In this way, we have found that for all epipedon classes it is possible to find a minimum, very low number of variables that when used, the elimination of the rest does not substantially compromise the accuracy of the method. This represents a big improvement, that has allowed us to obtain the relative relevance of the five types of variables reported in Table 1 for each epipedon, as shown in Fig. 6. Note that in this figure some observed relationships were a priori expected, e.g. an organic type *litos* is the most predominating factor in the organic class, this representing a further support of our method. The distributions obtained in the figure constitute a fingerprint—proxy—for each observable epipedon. Moreover, these quantitative refined relationships in Jenny’s Eq. (1) had not -at least to the authors’ knowledge- been reported before.

Notice that our SOM could have been used to produce a DSM^22,23^ of the geographical region under study and adjacent. This is certainly an interesting topic, but not directly related to the main aim of the paper, which deserves future research, in which issues such as uncertainties determination or validation of our dataset will have to be more specifically addressed.

In conclusion, starting from a null knowledge of both what variables should appear and what inter-relationships between them are relevant in Jenny’s relationship, in this work: (1) a minimum set of variables explaining the most common epipedons observed, and (2) the internal structure of the relationships between these variables, has been faithfully elucidated.

This study opens the door to further analyses that taking into account the results obtained here could investigate closed form relationships. Actually, our work represents in this sense a modest but important first step towards a true mathematical reinterpretation of Jenny’s seminal equation in the era of AI.

## Methods and auxiliary calculations

### Approach overview

In the data obtained in a soil field campaign, each observation point can be classified by one or more diagnostic horizons, which in turn can be either superficial (epipedons) or subsurface (endopedons), as shown in Fig. 1. A simplification process is required in both sides of Eq. (1), so that forming factors are then presented in the form of an aggregated set of (numerical and categorical) variables.

**Figure 8:**
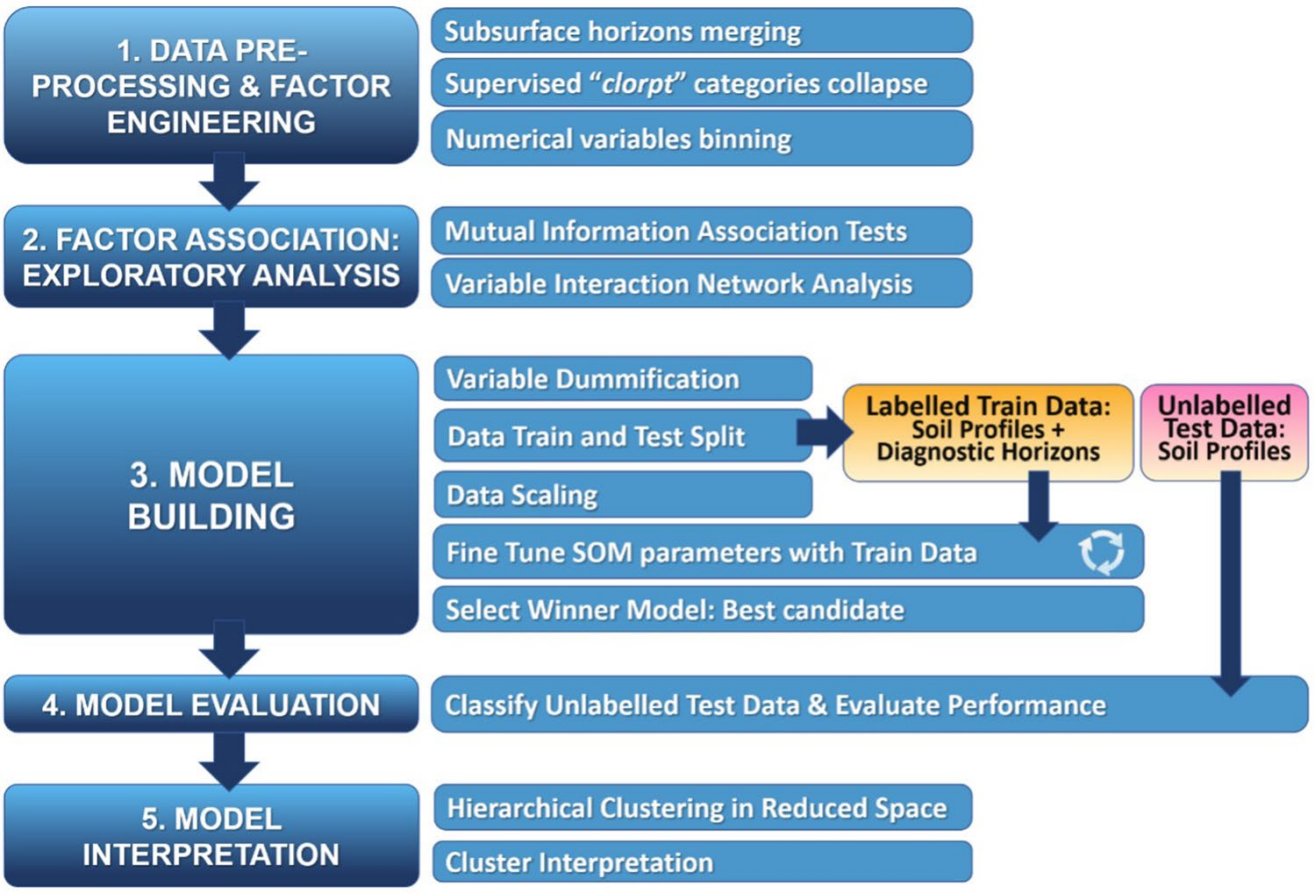
Methodological workflow. The left tier corresponds to the subsections in “Methods and auxiliary calculations”, and in the right one details of each one of them are given.

We summarize our methods in the workflow in Fig. 8. From a first data-preprocessing step, we implement a factor exploratory analysis by using information theory metrics. This allows to grasp the most informative features present in the data set. Then, feeding that subset into a Self-Organising Map (SOM) we find the most relevant existing clusters. To find the best model representing the data we next enforce a bootstrap cycle. The resulting model is then evaluated through its prediction power to classify unlabelled data. Finally, the resulting clustered SOM is interpreted in terms of the averaged quantities computed for each cluster. Each stage of the workflow is explained in more detail in the right tier of Fig. 8 and fully described in the following subsections.

### Data collecting and preprocessing

**Figure 9:**
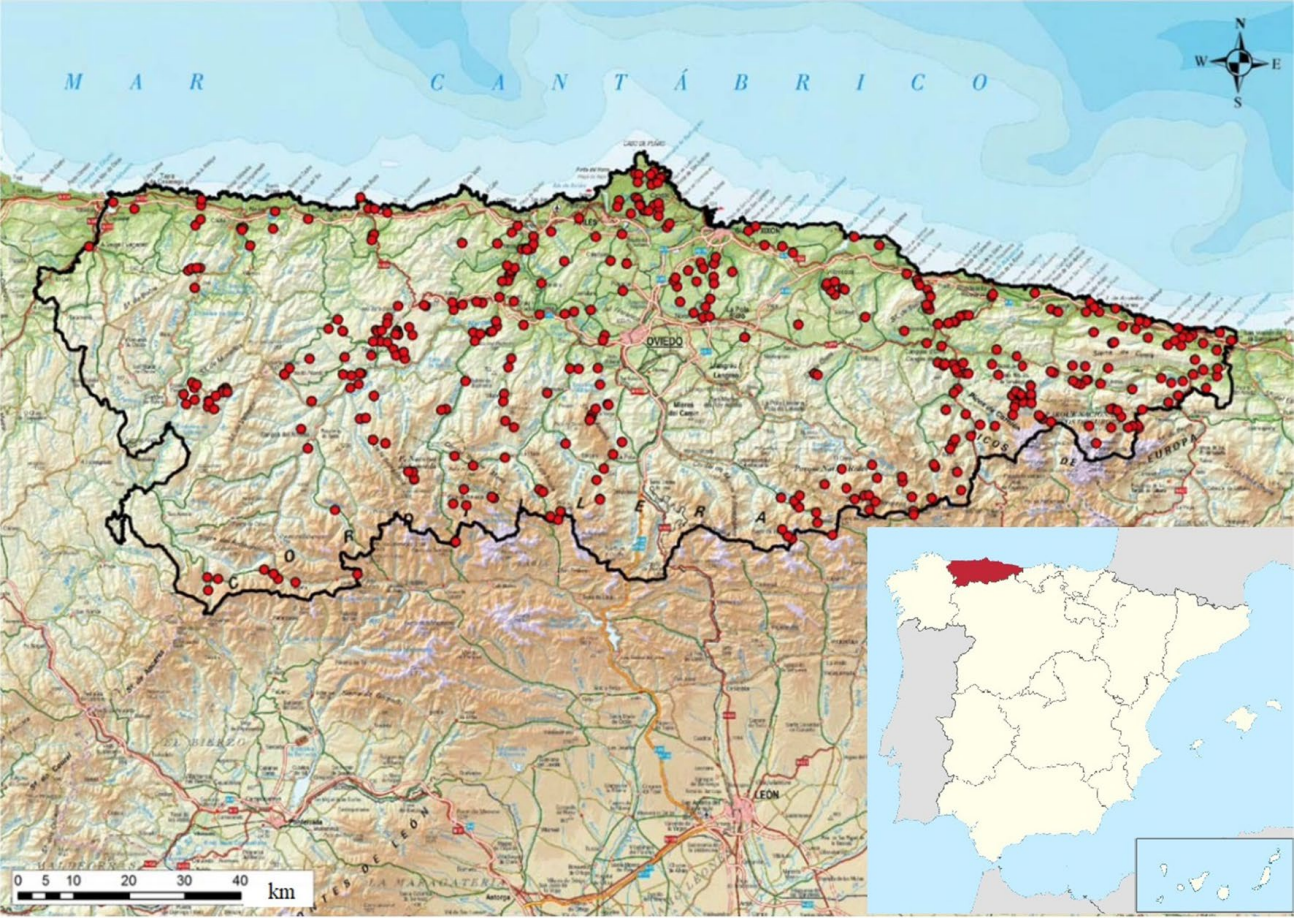
Localization in the Autonomous Community of the Principality of Asturias of the 442 sites visited during the 2001–2012 soil survey, where experimental values for the 15 variables listed in Table 1 were collected (Adapted from Fig. 5.1-01 of Ref. ^40^ https://repositorio.uam.es/handle/10486/671738).

The dataset used in this study consists on the experimental values for the variables listed in Table 1, that were collected in an extensive field study carried out in the period 2001–2012 at the 442 sites shown in Fig. 9 in the Autonomous Community of the Principality of Asturias, a coastal hilly region in the north of Spain. All the field work corresponded, essentially, to a physiographic type of sampling, based on lithological, geomorphological, and vegetation cover information. The variability of soils within each survey unit was determined by observations, usually made on cuts of the natural terrain, that were intended to cover the maximum possible range of altitudes. For a more detailed information, see Ref. ^40^.

For each prospection site, a record of 15 variables was obtained (see Table 1). The processing of the data (see right tier of Fig. 8) consists of the following steps:

1. Subsurface variables merging on the *S* component of Eq. (1),
2. supervised factor categories collapse on the *factors* component of the equation, and
3. binning of the numerical variables on the *factors* component of the equation.

As indicated above, for the purpose of studying the relationships between both sides of Eq. (1), only soil surface horizons are selected. However, in addition to these surface horizons, the soil as a whole is defined, in this case, with one or (not often) two subsurface horizons, labeled as *subsup*1 or *subsup*2, corresponding to albic, argillic, calcic, cambic, or spodic types^14^. The absence of any subsurface horizon, indicated as ‘no’, is indeed frequent. Considering the irregular presence of horizons, and in order to obtain a better predictor for deep soil horizons, we further merge subsurface variables *subsup*1 and *subsup*2 into a single one *subsup*.

In addition, during the initial phase of the data analysis we found that some of our variables had categories occurring with few observations ($<10$ in some cases), while others appear over-dimensioned. This is due to the intrinsic heterogeneity in the soil spatial distribution, which is more evident in territories, such as the one studied here, with a high complexity of forming factors. Variables and subsequent categories are then chosen based on physiographical criteria, observed in the soil profile location, and whose importance in pedogenesis has been previously contrasted in the literature. The values of each category therefore come from *in situ* raised information on the 442 soil sampling points. All this leads to establish simplification criteria in order to adequately treat the abundant information generated on such points. This simplification has been carried out based on the minimization of the number of representative variables of each forming factor, as well as merging the categories that characterize them. Once the categories were merged, we transformed all the numerical variables into categories by a cutting procedure based on quartiles, so that each bin has approximately the same number of observations. Our experience determines that this way of proceeding is enough to obtain an adequate representation of the numerical variables.

### Factor association: exploratory analysis

Once the data has been pre-processed we want to know what relationships exist between the variables, and if so, how strong these interactions are. Due to the scarcity of data and the heterogeneous nature of the variables (mixture of numerical and categorical quantities), we approach this stage in two steps:

1. Fisher/Mutual Information (MI) association tests, and
2. variable interaction network analysis.

**Figure 10:**
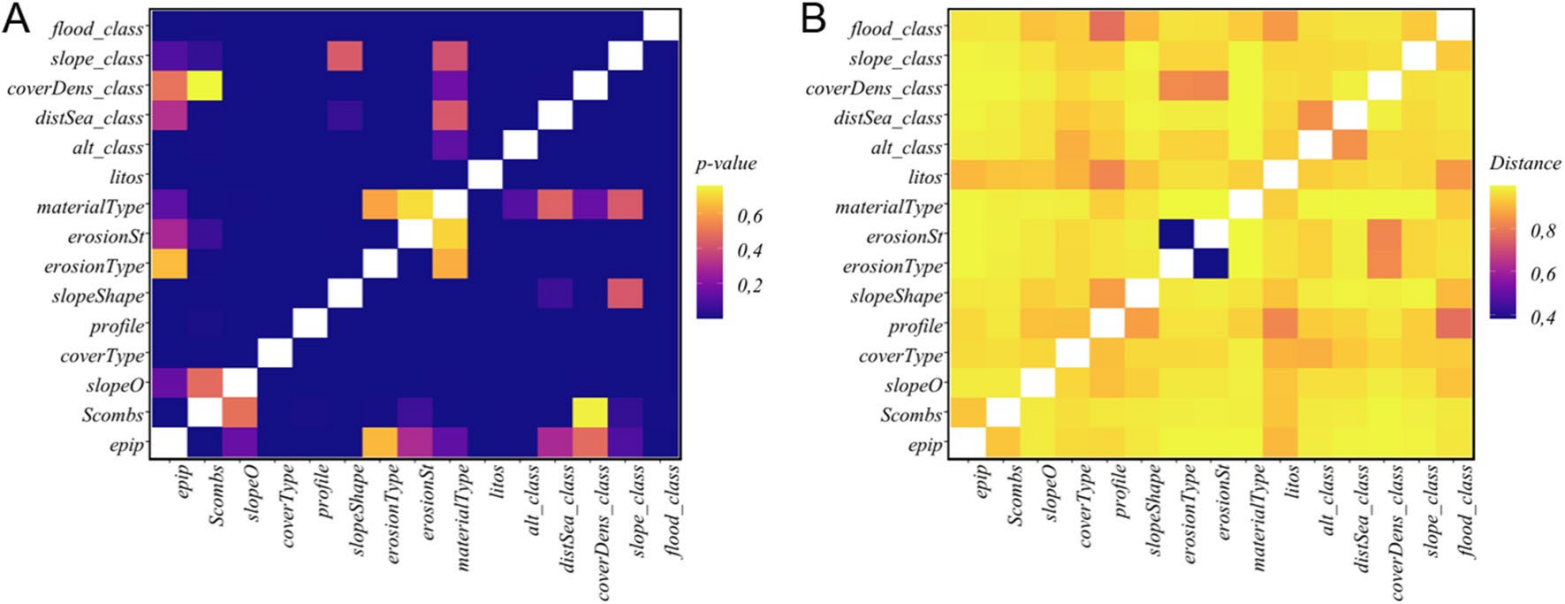
Factor association: comparison of the results obtained with Fisher’s *p*-value (**A**), and the metric introduced with Eq. (2) (**B**), for all possible pair of forming factor variables. These figures have been produced with the *ggplot2*^42^ package in the *R* programming language^43^..

First, we observe that even with the subsoil combination and level fusion techniques described above, some combinations of factors have less than 5 observations. Therefore, we have used an exact Fisher test to measure the strength of the correlation between pairs of variables. To do this, under the null hypothesis, i.e. independent variables, we use the *p*-value as a proxy for the strength of association: the lower the *p*-value, the greater the correlation. However, we observe that this procedure tends to overestimate the correlations, and it is not possible to distinguish association patterns between variables. For this reason, we have decided to use a different strategy based on information theory. We then use the expression given in^41^

2 $$\begin{aligned} d(X,Y) = 1-\frac{MI(X,Y)}{H(X,Y)} \end{aligned}$$

being *MI*(*X*, *Y*) the mutual information between variables *X* and *Y*, and *H*(*X*, *Y*) the associated Shannon entropy. In Fig. 10 we compare the results rendered by both metrics. (Notice that low values (blue) in *p* and high values (yellow) in *d* indicate high correlations.) As can be seen, the former metric predicts a mostly uniform highly correlated variables, with few interrelations. This is rather unusual and certainly not coherent with the results in Fig. 2. This lead us to the conclusion that *d*(*X*, *Y*) outperforms Fisher’s test *p*-value, since most false positive correlations detected in Fisher’s test are absent. Accordingly, we use the second criteria in the rest of the paper.

**Figure 11:**
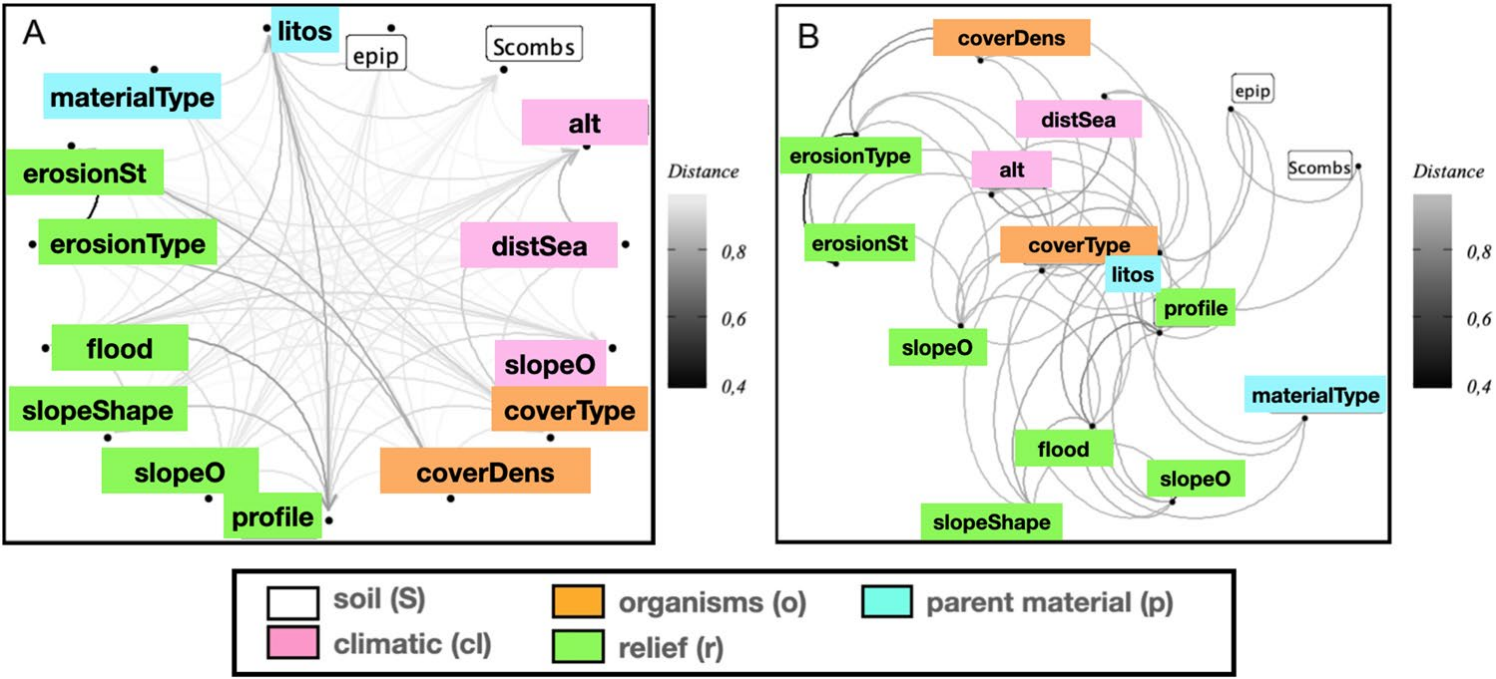
Factor association strength analysis: link weights are proportional to the inter-factor distance computed from Eq. (2): (**A**) complete graph, (**B**) reduced graph resulting from elimination of those links where the distance (2) between two nodes is above the median.

Finally, a better visualization is obtained from a directed network in which the links have a weight proportional to the distance given by the Eq. (2). In Fig. 11 we show the complete network and the reduced network resulting from considering only those links where the distance between variables is within the first quartile. After this step, the factors appear segregated into two related subgroups. The smallest one is associated to erosion and coverage density type variables, as it expected, while the greatest subnet shows how some variables like *profile*, *litos*, *coverType*, and *floodClass* are the important network connectors, i. e., hubs^3^. Once the network has been pruned, we use a community structure detection algorithm via short random walks^29^. This allows us to find the existing aggregations among the relevant observables/variables, which was discussed in “Results” in relation to Fig. 2.

The next step, according to our workflow in Fig. 8 is to build the model that best reproduces the joint probability distribution observed in the data.

### Model building

There are many techniques available for dimensionality reduction and data clustering. After experimenting with different methods, we have found that a self-organized multi-layer map (SOM) is very effective for our case. SOMs are a type of Artificial Neural Network (ANN) devised by Kohonen^20,32–34^, with an architecture and learning mechanism that imitates the specialization by areas (vision, smelling, etc.) taking place in the brain. When a dataset is presented to a SOM, the processing units (neurons) undergo a competition process resulting in a segmentation into different regions. These regions are adapted/specialized in subsets of data that are similar to each other (in terms of a metric distance). For a detailed description of the SOM learning process used by us see Appendix A in the SM.

The most interesting feature about the SOM strategy is that it produces a mapping from a high dimensional space into another of low dimensionality, preserving distances. As a result, observations which are close to each other in terms of distance belong to regions of the SOM that are also close to each other. SOMs are popular in the ML community for their application to unsupervised classification problems, since they allow to find relevant patterns in the data without having to specify *a priori* the number of classes (as it happens, for example, with the popular *k*-means). In this sense SOMs have been used in very diverse areas such as medicine, engineering^44^, and even social sciences^45^.

The construction of the optimal model (see general workflow scheme in Fig. 8) goes through the following steps:

1. Variable dummyfication (i.e., eliminate the effect of the variable by replacement with random values; but maintaining the dimensionality of the problem),
2. data training/test splitting,
3. data scaling,
4. fine tuning the SOM parameters,
5. select the winner model, and
6. classify unlabelled test data and evaluate performance.

In order to have a numerical representation of the variables in the soil-profile layer we use the technique known as *variable dummyfication*, which simply transforms each variable into a Boolean vector, 0 or 1, depending on whether the observation is in the class or not. Once *dummyfied*, the resulting data are split into 3/4 for training and 1/4 for model validation (see Fig. S2 in Appendix B of the SM). In addition, a scaling of the numerical layer is done so that the variables have mean 0 and variance 1. In this way, data with very different ranges and scales are more comparable. In the next step, the best possible SOM is obtained through data resampling and parameter optimisation. This is done by grid searching the SOM parameter space $\vec {\theta }$. By keeping the remaining SOM parameters fixed, we set $\eta =1-\lambda$. (For example, $\lambda =0.1$ means that the numerical component of the distances in Eq. (A1) of Appendix A in the SM is given a weight 9 times greater than that of the category component.) In our calculations, we have used a linear decreasing function $f_\alpha$ with slope $\alpha =0.05$, an hexagonal map topology, and a neighbourhood radius equal to the third quartile of the distances between units, and 100 learning periods. Then, for each tuple of parameters (point on the grid), 100 data sets are extracted at random with replacement from the training data. For each of these pieces, a SOM is trained with 3/4 of the data of that piece with the *SOM Training* algorithm 1 (see Appendix C in SM), following the scheme shown in Fig. S2(3)—Appendix B in SM—and using the generalized distance of the Eq. (A1) of Appendix A in SM. The remaining 1/4 of the data in the piece is mapped to the resulting SOM, finding the accuracy of the prediction, which are shown in Fig. S2(4) and (5) in Appendix B of SM. This is done using the *Classification and Accuracy* algorithm 2 (see Appendix C of SM).

This procedure is repeated for each sample, and the average performance across all hold-out predictions are calculated. The result is a performance level for this set of parameters. Using a grid search technique, the optimal set of parameters is found, and this defines the best possible model.

We stress that during the training phase, we have enforced a data-resampling through the *bootstrap* method, which outperforms the standard cross-validation in this case^46,47^. We hace used the algorithm of *boot632* available in R through the package Caret^48^. In Figs. S2 and  S3 of Appendix B in SM we show diagrams illustrating how the process is carried out to find the best model. This process takes place during phases 3 (Model Building) and 4 (Model Evaluation) of our workflow, that was presented in Fig. 8.

Once the best candidate model is found, the unlabelled test data is used to find the prediction accuracy. This is done by using again algorithm 2 in Appendix C in SM with the winning model. Results are shown in Fig. 3.

### Model evaluation and interpretation: coarse-graining the SOM

**Figure 12:**
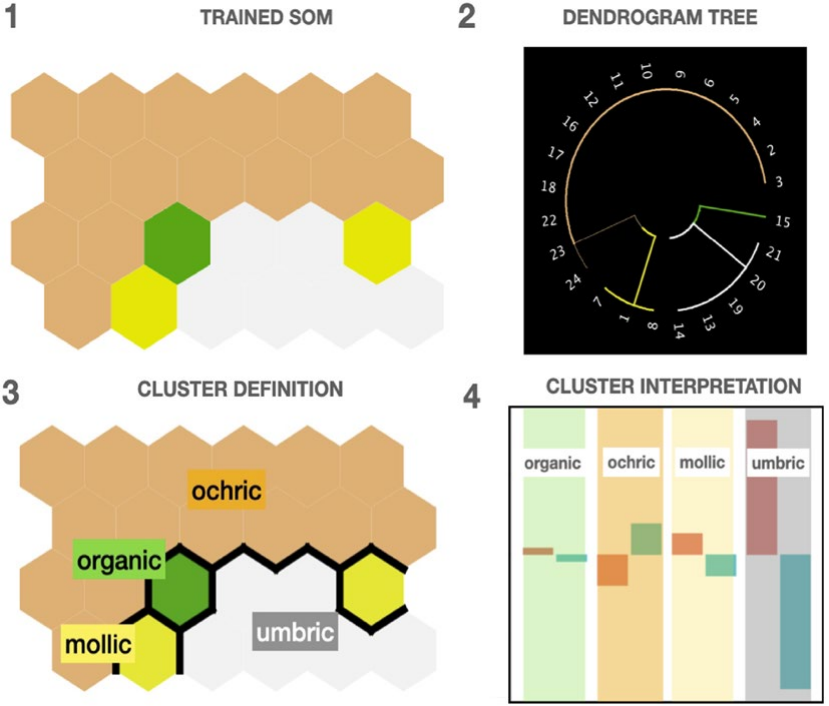
Cluster definition and interpretation in the SOM. (1) SOM soil-profile layer is coarse-grained through a hierarchical clustering in the diagnosis horizon layer. (2) The resulting dendrogram tree defines four clusters (3) corresponding to the mollic, ochric, umbric, and organic diagnostic horizons classes. (4) Each cluster, i. e., diagnostic horizon, can be then interpreted by the values of the soil-profile variables in its own units; results for *materialType* is shown, as an example.

To conclude with the SOM construction, we force a clustering (or coarse-graining) in the reduced SOM space^49^, using the procedure illustrated in Fig. 12. For this purpose, we use a hierarchical clustering of the (Tanimoto) distances among the unit codebooks in the diagnostic horizons layer (step 1 in the figure). The resulting *dendrogram tree*, depicted in step 2, clearly shows four clusters corresponding to the mollic, ochric, umbric, and organic diagnostic horizons (step 3).

One way to characterize each of these clusters is to look into the SOM codebooks containing the numerical information of the soil-profiles in each of the clusters. Then, by aggregating the codebooks corresponding to that factor over the units belonging to that cluster it is possible to find a coarser-grain description of the diagnostic horizons. By repeating this analysis for each of the factors, we obtain a *footprint* of the diagnostic horizon classes. For instance, in Fig. 12(4) the standardized mean values for the *materialType* variable are shown.

### Key forming factors detection

**Figure 13:**
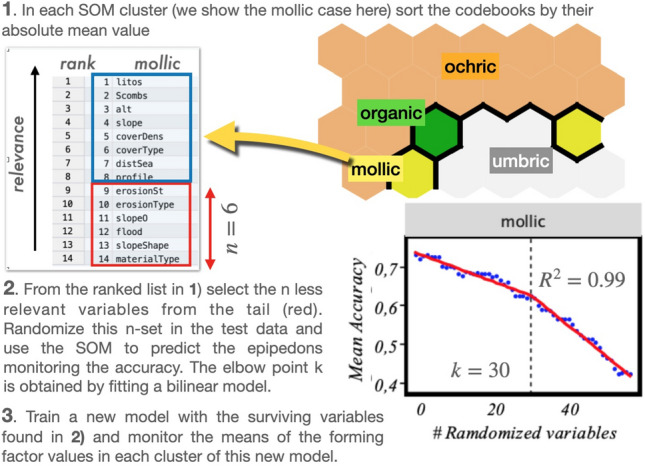
Procedure to determine the optimum minimum number of variables for each epipedon. The case of mollic is shown here.

As stressed in the last part of Results to refine our search for the key forming factors we opt for a sifting process in which we gradually eliminate the less important variables. The procedure is schematically shown in Fig. 13. We first, order the variables in a SOM cluster by their relative importance, considering their mean values in it (step 1). This allows to rank the variables for each epipedon according to their relevance. We then select the less significant ones, and monitor the global accuracy of our prediction by randomizing these set of variables—this being equivalent to eliminating them with increasing importance—, running at each step the model again to quantitatively gauge the resulting prediction accuracy (step 2). Finally, we train a new model with the surviving variables, and monitor the means of the forming factor values in each cluster of this new model (step 3). By assuming a bi-linear model^37^ it is possible to estimate the optimal number of randomization variables where the accuracy suffers a significant change.

### Supplementary Information

Supplementary Information.

## Data Availability

The datasets used and analysed during the current study available from the authors on reasonable request.
