# Supplementary material for: Disentangling Jenny’s equation by machine learning
Source: Sci Rep. 2023 Nov 27;13:20916. doi: 10.1038/s41598-023-44171-x (PMC10684535; doi:10.1038/s41598-023-44171-x)
Supplement: Supplementary file 1 — Supplementary Information. [file 41598_2023_44171_MOESM1_ESM.pdf]

Supplemental Material to *"Disentangling Jenny's Equation by Machine Learning"*

F. Prieto-Castrillo[1†], M. Rodríguez-Rastrero[2†], F. Yunta[3†], F. Borondo[4†] and J. Borondo[5,6*†]

[1]Departamento de Matemáticas, Universidad de Oviedo, Calle García Lorca 18, Oviedo, 33007, Principado de Asturias, Spain.
[2]Departamento de Medio Ambiente, Centro de Investigaciones Energéticas, Medioambientales y Tecnológicas (CIEMAT), Avenida Complutense 40, Madrid, 28040, Spain.
[3]Joint Research Centre (JRC), European Commission, Via Enrico Fermi 2749, Ispra, 21027, Italy.
[4]Departamento de Química, Universidad Autónoma de Madrid, Cantoblanco, 28049, Spain.
[5*]Departamento de Gestión Empresarial,Universidad Pontifícia de Comillas, Madrid, Spain.
[6*]AgrowingData, Almería, Spain.

*Corresponding author(s). E-mail(s): jborondo@gmail.com; Contributing authors: prietofrancisco@uniovi.es; manuel.rodriguezrastrero@ciemat.es; felipe.yunta-mezquita@ec.europe.eu; f.borondo@uam.es;
†These authors contributed equally to this work.

Abstract

In this Supplemental Material we present the five appendices referenced in the main text of the paper.

Keywords: machine learning, soil science, Jenny equation

Appendix A SOM Learning Specifics

In Fig. S1 we illustrate the training process of a SOM with a one-dimensional dataset consisting of colors. Basically a SOM specializes areas of your map in data (shown as colors in the example) through an adaptive process. The result is a clusterized map in which each cluster in the SOM represents a set of related observations. The distance between the unit weights, also known as *codes* or *codebooks* of each unit k in the SOM, and an observation x_j, $d(x_j, \omega_k)$ can be defined in several ways; for classifications of numerical data, the Euclidean distance is usually used. Moreover, the parameters defining a SOM are the following:

- unit network topology
- decreasing learning function f_α
- BMU neighbourhood radius
- learning epochs (number of times that the whole data is presented to the network)

With the scheme shown in Fig. S1 the SOM is normally used for unsupervised classification, since there is no way, in principle, to label the resulting clusters. However, it is possible to use SOMs also in a supervised way by including an additional layer of neurons. This multi-layer SOM (MLSOM or

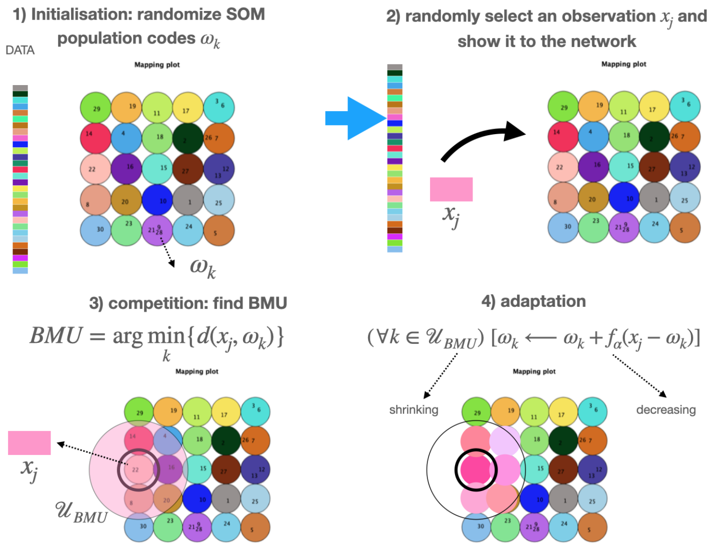

Fig. S1 Self-Organized Map training phase (schematic): 1) First, the map is initialized by assigning a random weight ω_k (color in the example) to each unit k, 2) then a randomly selected observation is presented to the map, and the best matching unit (BMU) is chosen, 3) every unit in the BMU neighborhood minimizes the difference between its weight and BMU's weight through a distance function, 4) then the process is iterated and both the learning function and the neighborhood decrease over time. A detailed description is found in Alg. 1

superSOM) allows to find classes when observations (soil profiles in our case) include a label (diagnostic horizons). In this case, a weighted distance over all layers is calculated to determine the winning units during training [1]

$$D(\vec{x}_j, \vec{\omega}_k) = \lambda d(x_{j1}, \ldots x_{jn-1}, \omega_{k1}, \ldots \omega_{kn-1}) + \eta h(x_{jn}, \omega_{kn}), \qquad \text{(A1)}$$

where $\lambda \in \mathbb{R}$ and $\eta \in \mathbb{R}$ are two weights determining how the numerical and categorical layers contribute to the distance. Usually, the Euclidean metric for $d(x_j, \omega_k)$ and the Tanimoto distance for $h(x_j, \omega_k)$ are used, as it is done in the present work.

Appendix B Model Tuning Schematic

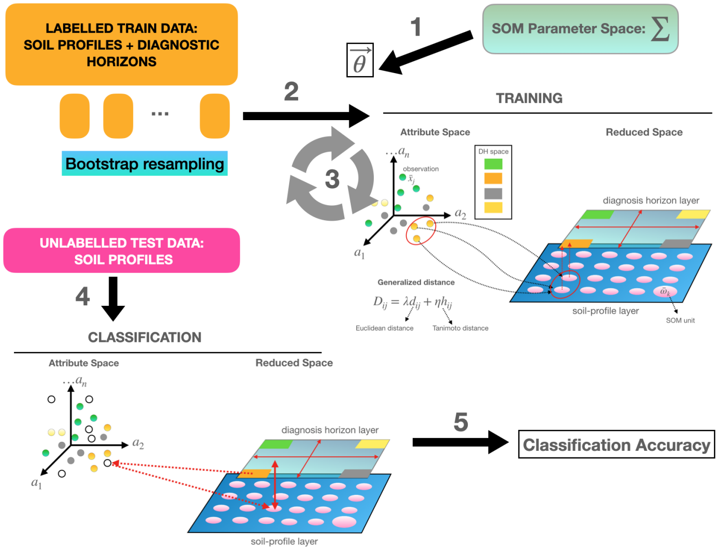

Fig. S2 Best model tuning and classification. For each SOM parameter vector combination θ, (1), 100 data sets are extracted at random being replaced with data from the training set. Each sample is used to train a SOM with 3/4 of the data of that piece, using the generalised distance (A1), (2). The remaining 1/4 of the data in the piece is mapped to the resulting SOM, finding the accuracy of the prediction. This is repeated, (3), for each sample and the average performance across all hold-out predictions are calculated (details in Fig. S3). Using a grid search technique, the optimal set of parameters is found. Finally, the unlabelled test data, (4) is used to find the prediction accuracy of the winner model, (5).

In the training phase of this workflow -steps 2) and 3)- we implement data-resampling through the *bootstrap* method described in Fig. S3.

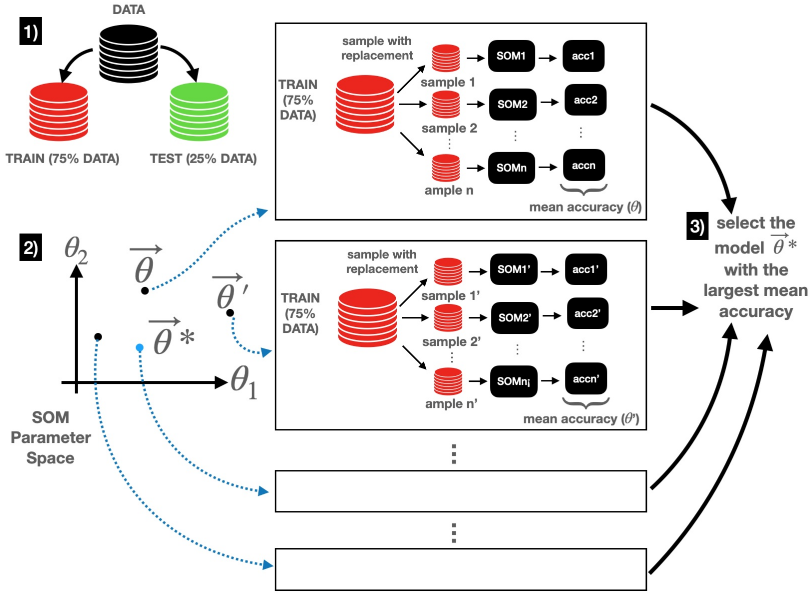

Fig. S3 Data re-sampling in the training phase. First the data is divided into training and testing chunks 1). Then, for each model parameter combination, n samples are obtained by shuffling the training piece 2). For each parameter, the mean accuracy is computed over the corresponding models ($acc1, \ldots, accn$) and the winner model is selected 3).

Appendix C Algorithms

In this section we present the two algorithms used for training and classification of the SOM.

Algorithm 1 SOM Training

INPUT: labelled data, SOM parameters
OUTPUT: two-layered trained SOM
procedure TRAIN
  $\vec{\omega} \leftarrow$ (randomize SOM population codes)
  repeat
    for all observations **do**
      $\vec{x}_j \leftarrow$ randomly select a DH-labeled soil-profile (observation)
      overall competition: chose BMU (euclidean+Tanimoto dist.)
      local collaboration: BMU diffuses knowledge among its peers
      adaptation: each BMU peer minimizes its discrepancy with the BMU by adapting its code
    end for
  until stop criterion reached
end procedure

Algorithm 2 Classification and Accuracy

INPUT: labelled data, 2-layer trained SOM
OUTPUT: classified data, Accuracy
procedure MAPPING
    map (find BMU) for new observation using numerical layers (euclidean distance)
    assign the class with highest proportion in the mapped unit
    repeat for each new observation
end procedure
procedure ACC
    compute accuracy from confusion matrix of real and predicted labels
end procedure

Appendix D Partial Correlation Analysis of Forming Factors

Finally, to further check our analysis of the importance of the formative factors, we have included a partial correlation study. In Fig. S4 we show the partial correlations for each pair of formative factors controlling for all others. As we have categorical variables, we again use the *dummification* technique as we have done previously. The first thing that is striking in this matrix is that there are

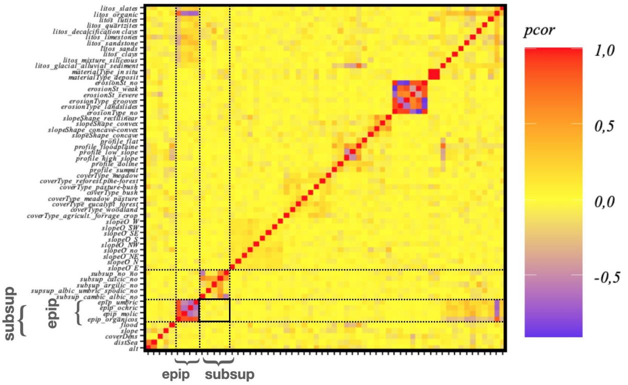

Fig. S4 Partial correlations among the dummy variable version of the forming factors. We have used Pearson's method implemented in the *pcor* library of *R* with p-values always less than 10^{-5}. There are no significant partial correlations between the group of *epip* variables and the group of *subsup* variables (black rectangle)

no significant partial correlations between the group of epip variables and the group of subsup variables (black rectangle marked in the figure). However, our network analysis (see Fig.2 in main text) was able to establish a relationship between epipedon and subsoil factors.

To find the associations between the original variables we do the following

(see Fig. S5). For each group (e.g. *litos*) we take the maximum absolute value of the partial correlation *pcor* outside its off-diagonal group and identify the corresponding original variable. As a result of this method, we obtain the

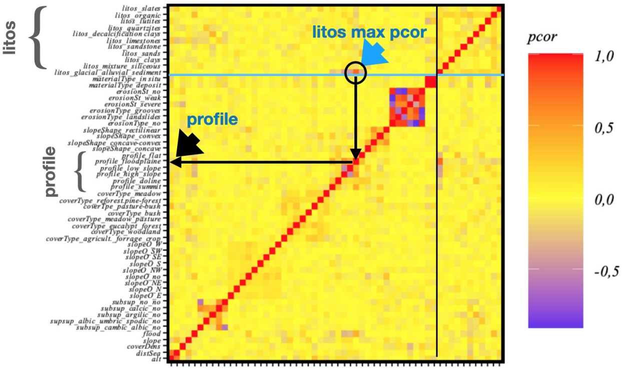

Fig. S5 Illustration of how we obtain the partial correlations for the original variables from the values obtained using *dummified* variables. For each group (e.g. litos) we take the maximum absolute value of the partial correlation *pcor* outside its off-diagonal group and identify the corresponding original variable

most important partial correlations between the training factors as shown in Fig. S6. In view of the results of this figure we see that, with the exception

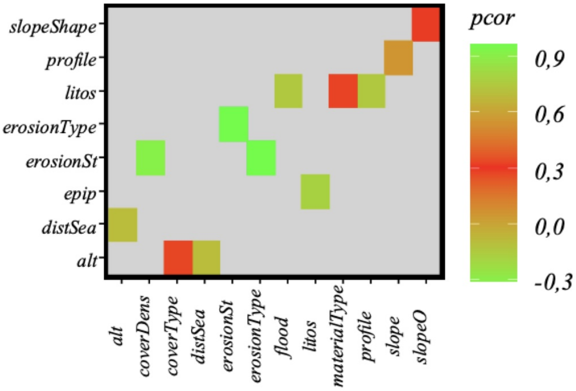

Fig. S6 Partial correlation values among the forming factors. Note that the matrix is not symmetric as a result of the method we have used to derive the *pcor* values. (see Fig. S5). Correlations between *epip* and *subsup* are not shown as they are not significant. With the exception of the relationship between slope and profile, the correlations shown here are all contained in the network analysis (Fig.2 in main text).

of the relationship between slope and profile, the correlations shown here are all contained in the network analysis (Fig.2 in main text). On the other hand,

the fact noted above that the partial correlation analysis does not detect an important association such as *epip/subsup* seems to be a serious limitation. For these reasons, we conclude that our network analysis outperforms the partial correlation analysis.

Appendix E Shapley Analysis of Forming Factors

To complement our study, we have carried out an analysis using the Shapley values method [2, 3], which allows to estimate the relative contributions of each of the factors. In short, Shapley values average the effect of including or not including a certain factor in the final result, taking into account all the possible *coalitions* -i.e. combinations- of that factor with the rest. Shapley's method comes from game theory, and has been successfully applied in various fields [3]. In Fig. S7 we show the results of the Shapley value analysis applied to our problem. We notice that Shapley's method predicts that the factors

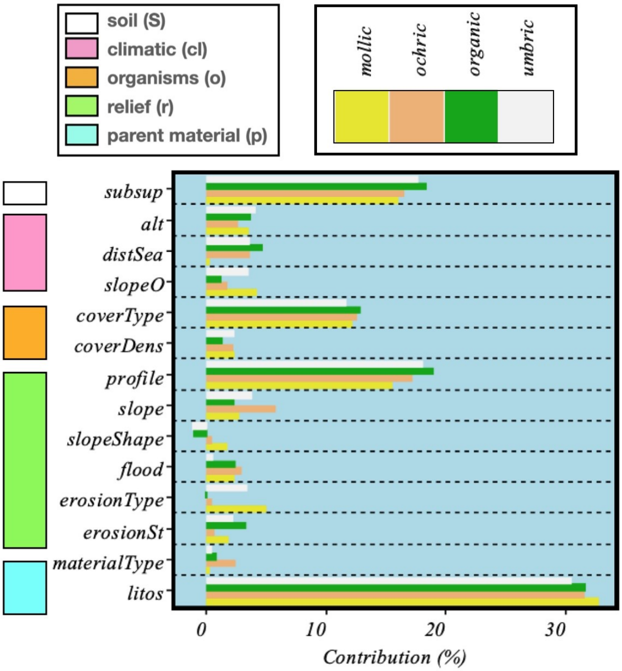

Fig. S7 Shapley Value Analysis of the forming factors. For each epipedon, the relative contribution of each forming factor is shown. Factors are also grouped as in Figs. ?? and ?? to improve interpretation.

lithos (parent material), *profile* (relief), *coverType* (organisms) and soil combinations (*subsup*) are the main contributors to explain the model. Note that these contributions are very similar for each epipedon. In other words, Shapley

values fail to discern well between different epipedons. On the other hand, our iterative method is more refined because it allows to isolate, for example, that *litos* (with organic value) is much more significant for the organic epipedon than for the rest of the epipedons (Fig. 6 in the main text). Furthermore, our method allows to distinguish not only which variables are important but also which values of these variables are important.

Acknowledgements

This work has been partially supported by the Spanish Ministry of Science, Innovation and Universities, Gobierno de España, under Contract No. PID2021-122711NB-C21.
The authors wish to thank Ricardo Pérez-Ochoa (Government of Asturias) and José Gumuzzio, for facilitating access to basic soil information, and Javier Rodríguez Alonso (INIA, Madrid) for managing georeferenced data.

References

[1] Wehrens, R., Kruisselbrink, J.: Flexible self-organizing maps in Kohonen 3.0. J. Stat. Softw. **87**(7), 1–18 (2018). https://doi.org/10.18637/jss.v087.i07

[2] Lundberg, S.M., Lee, S.-I.: A unified approach to interpreting model predictions. In: Proceedings of the 31st International Conference on Neural Information Processing Systems. NIPS'17, pp. 4768–4777. Curran Associates Inc., Red Hook, NY, USA (2017)

[3] Aas, K., Jullum, M., Løland, A.: Explaining individual predictions when features are dependent: More accurate approximations to Shapley values. Artificial Intelligence **298**, 103502 (2021). https://doi.org/10.1016/j.artint.2021.103502
